# Zebrafish as a Model for the Study of Lipid-Lowering Drug-Induced Myopathies

**DOI:** 10.3390/ijms22115654

**Published:** 2021-05-26

**Authors:** Magda Dubińska-Magiera, Marta Migocka-Patrzałek, Damian Lewandowski, Małgorzata Daczewska, Krzysztof Jagla

**Affiliations:** 1 Department of Animal Developmental Biology, Faculty of Biological Sciences, University of Wrocław, Sienkiewicza 21, 50-335 Wrocław, Poland; magda.dubinska-magiera@uwr.edu.pl (M.D.-M.); marta.migocka-patrzalek@uwr.edu.pl (M.M.-P.); damian.lewandowski@uwr.edu.pl (D.L.); 2 Genetics Reproduction and Development Institute (iGReD), INSERM 1103, CNRS 6293, University of Clermont Auvergne, 28 Place Henri Dunant, 63001 Clermont-Ferrand, France

**Keywords:** statins, fibrates, ezetimibe, zebrafish, muscle, myotoxicity, side effects of hyperlipidaemia treatment

## Abstract

Drug-induced myopathies are classified as acquired myopathies caused by exogenous factors. These pathological conditions develop in patients without muscle disease and are triggered by a variety of medicaments, including lipid-lowering drugs (LLDs) such as statins, fibrates, and ezetimibe. Here we summarise the current knowledge gained via studies conducted using various models, such as cell lines and mammalian models, and compare them with the results obtained in zebrafish (*Danio rerio*) studies. Zebrafish have proven to be an excellent research tool for studying dyslipidaemias as a model of these pathological conditions. This system enables in-vivo characterization of drug and gene candidates to further the understanding of disease aetiology and develop new therapeutic strategies. Our review also considers important environmental issues arising from the indiscriminate use of LLDs worldwide. The widespread use and importance of drugs such as statins and fibrates justify the need for the meticulous study of their mechanism of action and the side effects they cause.

## 1. Introduction

Drug-induced myopathies are classified as acquired myopathies caused by exogenous factors. These pathological conditions develop in patients without muscle disease and are triggered by a variety of medicaments, including lipid-lowering drugs (LLDs), neuroleptics, anticancer agents, antibiotics, corticosteroids, antivirals, and many others [1,2,3]. Drug-induced myopathies arise as a side effect of drug therapy intended to target a medical condition not directly related to muscle symptoms. These kinds of myopathies are manifested by muscle disorders which can be defined by the common term myotoxicity. This problem affects many groups of patients and its consequences could be fatal.

The term drug-induced myopathy is very broad, covering an extensive spectrum of symptoms ranging from myalgia (muscle pain or weakness without creatine kinase, CK, elevation), myositis (muscular complaints with CK elevation) to extremely serious symptoms associated with necrosis or rhabdomyolysis. The mechanisms underlying drug-induced myopathies are very diverse and can include direct muscle damage caused by mitochondrial injury or immune-mediated inflammatory damage [4].

Hyperlipidaemia is the most common dyslipidaemia. It is a pathological condition manifested by abnormal amounts of lipids (e.g., triglycerides, cholesterol, fatty phospholipids). Hyperlipidaemia is defined as abnormally elevated levels of any or all lipids and lipoproteins in the blood. This includes hypercholesterolaemia characterized by high cholesterol levels in the patient’s blood. Dyslipidaemia therapies are based on the use of various LLDs which include statins, fibrates, niacin, bile acid sequestrants, ezetimibe, lomitapide, phytosterols, omega-3 supplements, and PCSK9 inhibitors [5,6]. Widely used LLDs, intended to reduce the risk of cardiovascular diseases (CVD), have been the most commonly reported drugs to be associated with the adverse effects manifested by myotoxicity [3,7].

This manuscript provides a summary of the current understanding of myopathy induced by LLD therapy, as a serious and common side effect of treatments used in hyperlipidaemia. It describes data gained via studies conducted using various models, such as cell lines and mammalian models, and compares them with the results obtained in zebrafish (*Danio rerio*) studies (Table 1). The article also includes considerations on the directions of further research on LLDs and myopathies induced by their use, as well as the possibilities of using zebrafish in such studies. The deliberations presented here go beyond studies directly focused on issues related to the effects of LLDs on target organisms. Our review also considers important environmental issues arising from the indiscriminate use of these therapeutics and their influence on non-target organisms.

## 2. Pathological Mechanisms Underlying Lipid-Lowering Drug-Induced Myopathies

### 2.1. Statins

The pharmacological function of statins (e.g., simvastatin, SIM, and atorvastatin, ATV) is the inhibition of 3-hydroxy-3-methylglutaryl coenzyme A reductase (HMGCR), which is the key enzyme of cholesterol synthesis in the mevalonate pathway in the liver (PubChem compound identification numbers are summarized in Table S1). The inhibition of HMGCR has an impact on the intermediates of cholesterol synthesis such as coenzyme Q10 (CoQ10; ubiquinone), geranylgeranyl pyrophosphate (GGPP), and farnesyl pyrophosphate (FPP) [8,9,10].

Recent studies revealed that statins, despite lowering the cholesterol level, could have additional benefits, e.g., anti-inflammatory, antioxidant and immunomodulatory effects, inhibition of platelet activation, regulation of pyroptosis (a highly inflammatory form of lytic programmed cell death), increase in plaque stability, and improvement of renal function [11,12,13,14,15,16,17]. Nevertheless, some of the patients experienced side effects connected with muscle symptoms. It has been shown that there are several mechanisms involved in statin-induced myopathies.

It was suggested that in endothelial cells the function of the membrane-bound proteins could be changed after SIM treatment because the accompanying decrease in the level of cellular cholesterol, leading to increased cell membrane fluidity (since cholesterol is an important component of the cell membrane) [18,19]. The membrane fluidity changes and modification of muscle susceptibility occur as a result of the statin-dependent reduction of cholesterol. Modifications of membrane structure can influence the function of sodium, potassium, and chloride channels, resulting in muscle cell damage and leading to myopathies [20]. For example, studies on L6 rat myoblasts showed that SIM impaired the function of Na^+^/K^+^ and Na^+^/Ca^2+^ ATPase, which are crucial for maintaining the cellular membrane electrical potential [21].

Impaired prenylation (post-translational modification of protein requiring intermediates of the cholesterol biosynthesis pathway, which can be inhibited by statins) disturbs proteins’ ability to anchoring to membranes, leading to their deactivation [22,23,24]. Prenylated proteins are translocated from the cytoplasm to cellular membranes, where they interact with other important proteins. Almost 2% of cellular proteins undergo the covalent attachment of prenoid lipid adducts, farnesyl or geranylgeranyl [25,26,27,28]. Therefore, impaired protein prenylation might be a potential statin-induced myotoxicity mechanism (reviewed by [8]). Many proteins, e.g., lamins and small GTP-binding proteins (such as Rab, Ras, and Rho), are substrates for prenylation [29]. For example, prenylated Ras is involved in cellular proliferation and adhesion. Mullen et al. (2010) reported that disruption of cholesterol synthesis by SIM did not change the cellular CoQ10 level [30]. However, the Ras prenylation by geranylgeranyl pyrophosphate was decreased. Since only geranylated Ras can be attached to the sarcolemma of muscle cells, where it plays an important role in cell survival, inhibition of this process could explain the myotoxic mechanism of statins [31], reviewed by [8]. Additionally, ATV decreases the cholesterol level in C2C12 cells, which impairs the translocation and function of the glucose transporter GLUT4 [32], reviewed by [8]. Statin-induced inhibition of dolichols, other intermediates of the mevalonate pathway involved in protein N-glycosylation, may also lead to myopathy [20,33,34].

The observations of a decreased level of CoQ10 in statin-treated skeletal muscle of human patients and rodent models had led to the conclusion that statins may influence mitochondrial function, affect muscle function and disrupt its morphology [35,36]. Based on these studies, additional mechanisms of statin-induced myopathy can be directly or indirectly linked to mitochondria. The significant decrease of CoQ10 level, which is a key electron transporter localized in the inner membrane of mitochondria, and caused by lower cholesterol level, might result in the reduction of ATP production and cell damage [37], as reviewed by [8]. A decreased CoQ10 level in skeletal muscle in statin-treated patients and higher lactate/pyruvate ratio are considered as indicators of abnormal mitochondrial function [36,38,39].

Recent studies revealed that statin treatment in rats can cause mitochondrial membrane depolarization and a calcium wave (a localized increase of Ca^2+^) in skeletal muscle sarcoplasm with subsequent calcium release from the sarcoplasmic reticulum (SR). Ca^2+^ outflow from SR (so-called Ca^2+^ sparks) is a result of dissociation of ryanodine receptor 1 (RyR1), a process which is involved in pro-apoptotic signalling [36,40]. Numerous studies showed that an increased level of Ca^2+^ in cells is a major factor in apoptosis. Therefore, calcium regulation mechanisms, such as this mediated by RyR1, can be a crucial player in the regulation of apoptosis [41,42,43,44]. In contrast, no Ca^2+^ sparks were observed in cardiac myocytes after statin treatment, the reason being that RyR2, not RyR1, is present in cardiomyocytes [36,40,45].

It worth to noting that positive family history is a common risk factor for statin intolerance, as reviewed by [46]. Several studies show a significant association between muscle pain and single nucleotide polymorphisms (SNPs) in patients who have received statin treatments (e.g., SIM, and cerivastatin, CER) [47,48,49,50,51,52], as reviewed by [46]. For example, genome-wide scans of patients have revealed a strong association between SIM-induced myopathy and c.521T>C [53]. This association is a result of a nearly complete linkage imbalance between non-coding SNP in the *SLO1B1* and c.521T>C SNP linked with a reduction in the cholesterol-lowering effect of SIM, reviewed by [54]. These data suggested that statin-related myopathy may be a result of different genetic mechanisms [47].

In general, statins are well tolerated by patients. However, muscle-related side effects of statin treatment are frequently reported. Interestingly, one of the rare statin-associated side effects is immune-mediated necrotizing myopathy (IMNM) [55], reviewed by [56]. IMNM is diagnosed by the presence of muscle fibres necrosis and degeneration, and is correlated with CK level and muscle strength [56,57]. Additionally, based on presence of myositis-specific antibodies (MSAs), IMNM can be divided into: (1) anti-HMGCR, (2) anti-SRP (signal recognition particle), and (3) antibody-negative myopathy [58].

The pathogenesis of anti-HMGCR myopathy is poorly understood. Nevertheless, it has been proposed that statins could initiate autoimmunity by increasing the expression and availability of the autoantigen HMGCR. Additionally, statins could bind to HMGCR, causing its conformational changes, and alternate processing by antigen-presenting cells [59,60,61,62] reviewed by [56]. The in-vitro studies showed that anti-HMGCR and anti-SRP autoantibodies are involved in atrophy of muscle fibres and increase the *MAFbx* (muscle atrophy F-box; atrogin-1) and *Trim63* (*MuRF1*; *muscle RING finger 1*) transcription. Additionally, high levels of the inflammatory cytokines TNF and IL-6 were associated with IMNM. Furthermore, the observed decreased production of IL-4 and IL-13 resulted in impaired myoblast fusion [63].

### 2.2. Fibrates

Other LLDs, fibrates (e.g., fenofibrate, and clofibrate), decrease plasma triglyceride and cholesterol levels via several mechanisms comprising induction of lipoprotein lipolysis, stimulation of cellular fatty acid uptake, and reduction of triglyceride synthesis in the liver (Table S1). They can also increase the removal of low-density lipoprotein (LDL) particles, and lower neutral lipid exchange between very-low-density (VLDL) and high-density (HDL) lipoproteins. They also increase HDL level and stimulate reverse cholesterol transport [64].

Fenofibrate and clofibrate, agonists of peroxisome proliferator-activated receptor α (PPARα), are currently used to treat dyslipidaemia. However, treatment using a highly selective PPARα inhibitor in rats could result in skeletal muscle degeneration and necrosis [65,66]. The inhibition of the PPARα receptor by fenofibrate leads to increased β-oxidation and oxidative stress in skeletal muscle, as a consequence [66,67]. Furthermore, the additional oxidative stress may also result in mitochondrial dysfunction, because fenofibrate inhibits complex I (the first protein complex of the respiratory chain, which catalyses the transfer of electrons from nicotinamide adenine dinucleotide phosphate (NADPH) to CoQ10) [68]. A study by Pettersen et al. (2012) revealed that skeletal muscle degeneration correlates with increase in acyl-CoA oxidase (AOX) mRNA expression, which is involved in the PPARα signalling pathway. Surprisingly, no correlative increase of palmitoyl-CoA β-oxidation was detected. The histological analysis showed that necrosis took place only in type I muscle fibres. In these muscles, β-oxidation is the source of the energy; therefore its inhibition by fibrates leads to cell death [69].

The fibrate-induced skeletal muscle myotoxicity mechanisms involve the activation of PDK4 (pyruvate dehydrogenase kinase 4). Fibrates (e.g., bezafibrate, clofibrate, and ciprofibrate) inactivate pyruvate dehydrogenase complex, which is responsible for catalysis of irreversible decarboxylation of pyruvate to acetyl-CoA. As a result, limited oxidation of glucose and three-carbon compounds, and enhanced fatty acid oxidation are observed in cells [70,71]. One of the fibrates, gemfibrozil, has been suggested to inhibit the glucuronidation pathway, which increases the risk of muscle disorders. Furthermore, increased risk of muscle injury and fibrate concentration could result from another gemfibrozil-mediated pathway by inhibition of cytochrome P_450_2C8 (CYP2C8) activity [72,73]. Other fibrates, such as fenofibrate, have not demonstrated a significant effect on glucuronidation [74,75]. Furthermore, fenofibrate has poor potential to inhibit cytochrome P_450_3A4 (CYP3A4). However, fenofibrate acid (FA), which is a substrate of CYP3A4, has been reported to inhibit organic anion transporting polypeptide 1B1 (OATP1B1) [74,76,77,78].

### 2.3. Ezetimibe

Ezetimibe, like statins and fibrates, is successfully used to treat hypercholesterolaemia (Table S1). Ezetimibe’s mechanism of action is the selective inhibition of the cholesterol absorption in the intestine by blocking Niemann-Pick C1-Like 1 (NPC1L1) protein transporter, which is present in the enterocyte membrane and plays a key role in cholesterol uptake [79,80,81]. Numerous studies have revealed that ezetimibe, contrary to statins and fibrates, has not been associated with an increased rate of myopathy or rhabdomyolysis, whether used alone or in a combination with statins, as reviewed by [81]. Nevertheless, there have been several case reports of myopathy attributed to ezetimibe, where patients have had an elevated level of CK activity [82,83,84,85,86]. It is known that statins are hydrolysed by cytochrome P_450_ and metabolised by glucuronidation. Since ezetimibe is not metabolised by cytochrome P_450_ and it is extensively glucuronidated, it was proposed that impaired ezetimibe glucuronidation might be responsible for myopathy [87]. Hsiang et al. (1999) suggested that ezetimibe, like statins, might be a substrate for organic anion transporter 2 (OATP2) [88]. Case studies showed that ezetimibe in monotherapy can induce myalgia in patients who suffer from statin-induced myopathy. Ezetimibe could impair fatty acid oxidation as the possible pathology mechanism [84].

## 3. Models for Study of Lipid-Lowering Drug-Induced Myopathies

The LLD-induced myotoxicity assessments were conducted using a variety of models including cell lines and mammalian organisms (e.g., mouse, rat, goat, rabbit, and dog) (Table 1). Among various LLDs, statins are the most commonly applied in humans for the prevention and treatment of CVD. Therefore this group of drugs gain special attention from researchers [89].

In-vitro research using human and murine cell lines provides new data concerning LLD-induced myotoxicity and confirmed differences in the side effects caused by LLDs belonging to the same group (e.g., statins). This kind of research also helps to examine compounds which could protect cells exposed to LLD treatment. For example, recent studies carried out on mouse C2C12 skeletal muscle cells shed more light on the molecular mechanisms of the cytoprotective effect of geranylgeraniol (GGOH), a mevalonate-derived isoprenoid. GGOH protects cells treated with statins, precisely ATV and SIM, through the inhibition of calpains, which are calcium-dependent, nonlysosomal cysteine proteases [90]. Moreover, these experiments revealed that different statins, depending on the degree of their lipophilicity, cause more (SIM, higher lipophilicity) or less (ATV, lower lipophilicity) myotoxicity manifested in impaired cellular mitochondrial respiration. Notably, studies conducted on a rodent model of statin-induced myalgia have also confirmed that the administration of GGOH can prevent skeletal muscle fatigue [91].

Studies on cell lines have also provided a lot of valuable information regarding the molecular mechanism of LLDs’ mechanism of action, which has proven to be very complex. Specifically, primary human muscle cells exposed to a lipophilic SIM and hydrophilic rosuvastatin (RSV) display various changes in their metabolism, and gene and protein expression profiles (regarding more than 1800 mRNA transcripts and 900 proteins). In addition to its well-documented effects on cholesterol biosynthesis, treatment with both investigated statins causes changes in profiles of eicosanoids secreted by human muscle cells. It also disrupts their proliferation and differentiation. Furthermore, results of the study support the hypothesis that supplementation with omega-n fatty acids (eicosanoids precursors) might be beneficial as a prevention or as a treatment for patients undergoing statin therapy [92].

To meet the needs arising from the necessity to study LLD-induced myotoxicity, researchers continue to refine existing tools and develop new ones. An example is a microphysiological system based on patient-derived myoblasts. The cells form engineered myobundles mimicking the organization and function of native skeletal muscle, allowing for the study of skeletal muscle ex vivo development [93]. The system was used to investigate the statin-associated musculoskeletal symptoms [94]. Statin exposure leads to myotoxicity manifested in the reduction of cells’ contractile force, and disruption of sarcomeric actinin organization.

Data gained via in-vitro experiments, despite their undisputed advantages, are limited in terms of predicting in-vivo conditions and are not able to replicate the behaviour of cells in an entire living organism. Therefore, in-vivo studies conducted on more complex model organisms such as a mouse are thought to provide more valuable and reliable information regarding the effects of progression of particular diseases, and their treatment. Osaki et al. (2015) developed skeletal muscle-specific HMGCR knockout mice which were intended to mimic human post-statin myopathy conditions [95]. The generated model exhibited severe myopathy caused by the deficiency of HMGCR enzyme activity and resulting in depletion of mevalonic acid (MVA). In HMGCR knockout mice, induction of skeletal muscle cell membrane damage, myofibrils necrosis, and an elevated serum CK level were observed. Oral administration of MVA revealed that the generated model was completely rescued [95].

As mentioned in the previous chapter, also genetic polymorphisms are risk factors for LLD-induced myotoxicity. Research related to this phenomenon was conducted using transgenic mouse models carrying different slow-channel congenital myasthenic syndrome (SCS) mutations [96]. The results demonstrated that one of the genetic variants of the nicotinic acetylcholine receptor (nAChR) could be related to the onset of statin-induced side effects. The nAChR is a transmembrane glycoprotein expressed in skeletal muscle at neuromuscular junctions (NMJs), which transduces the chemical signal necessary for muscle contraction. Studies revealed that mice expressing a mutant variant of nAChR (SNP rs137852808; αC418W) display impaired neuromuscular transmission upon ATV treatment. The study provides an important clue to explain one of the most common statin side effects regarding neuromuscular problems contributing to muscle pain or weakness [96].

The histopathological changes, comprising hypercontraction and fibre necrosis, in muscle exposed to statins have also been examined using a rat model. Studies on rats have confirmed the distinct susceptibility of skeletal muscle to damage caused by therapy with different statins (more severe in the case of lipophilic SIM and lovastatin (LOV) than hydrophilic pravastatin [PRA]). Moreover, it was also reported that young rats are more susceptible to statin-induced muscle damage than adults [97].

Further investigations conducted on female rats revealed that type II muscle fibres (primarily glycolytic and poor in mitochondria) are most vulnerable to muscle injury caused by statins [98]. However, other research groups using young male rats obtained contrary results. According to their outcomes, the CER-induced myotoxicity affects only type I, but not type II fibres [99]. This suggests that susceptibility to muscle-related side effects induced by LLD therapy depends on additional factors such as age and/or gender.

Studies carried out on a rat model also made it possible to establish some details regarding molecular mechanisms underlying statin-induced myopathy [100]. The obtained results showed that SIM down-regulates PI3k/Akt signalling, and up-regulates FOXO transcription factors. The latter is followed by an increase in the transcription of genes implicated in proteasomal- and lysosomal-mediated protein degradation, such as *MAFbx*. Studies also revealed impairment of carbohydrate oxidation, the occurrence of oxidative stress, inflammation, and increased plasma CK level. Muscle necrosis appeared in the group of animals exposed to the longest statin treatment [100].

Further, proteomic analyses using a rat model have provided valuable information on the effects of LLDs, represented by statins (ATV, and fluvastatin, FLV) and fibrates (fenofibrate), on the expression profiles of treated skeletal muscle [101]. The mentioned analyses focused on the expression levels of proteins crucial for skeletal muscle functions, such as proteins associated with energy production systems (including oxidative and glycolytic enzymes and CK), heat shock proteins (providing protection against oxidative stress), and proteins that are components of myofibrils. Proteomic examination demonstrated that all treatments induced a general tendency to down-regulation of protein expression. [101].

The rabbit is also one of the animal models used to study myotoxicity phenomena caused by LLD exposure. Studies in this species have confirmed data gained from other models and provided an interesting insight into the muscle pathology induced by LLDs [102,103]. Treatment with statins leads to necrosis and degeneration of rabbit muscle fibres. Ultrastructural examination allowed the accompanying changes to be described in more detail, revealing the presence of autophagic vacuoles and swollen mitochondria, as well as disruption of myofibrils and Z-bands [103].

The goat is gaining acceptance as an established model for biomedical studies and research with environmental relevance. This is mostly related to methane emissions caused by ruminants. Methane is one of the major greenhouse gases and its emission influences the climate. Its enteric formation is a by-product of the digestive process of ruminants and directly results from the activity of anaerobic bacteria. The reduction of methane emission is currently one of the significant challenges worldwide. Various measures are being used for this purpose, including LOV supplementation of animals, such as goats [104]. Therefore, due to the side effects caused by statins, the influence of these compounds on goat skeletal muscle began to be studied [105]. The histology studies revealed the occurrence of LOV-induced goat muscle damage correlated with increasing dosages. Moreover, the proteomic analysis showed that LOV triggers complex modifications to carbohydrate metabolism, energy production, and muscular system development [105]. This shows how important it is to evaluate side effects when studying the use of known substances in new models or for new purposes.

The dog has proven to be an excellent model corresponding to human diseases. Kawata and Yokoi (2019) carried out studies to explain the effects of LOV and fenofibrate on a dog’s skeletal muscles [78]. Oral co-administration of LOV and fenofibrate caused skeletal muscle injury. Similarly to other animals tested in this respect, in the skeletal muscles but not in cardiomyocytes, elevated levels of CK and necrosis of skeletal muscle fibres were observed. Also, the conducted research also provides an interesting implication for examination and validation of non-invasive biomarkers of clinical drug-induced side effects. One of the proposed biomarkers of LLD-induced skeletal muscle injury is an increased level of miR-1 in plasma. miR-1 is a representant of microRNA particles, which are small non-coding RNAs, characterized by high stability in blood and muscle expression pattern [78].

The use of a variety of established and reliable animal research models enables the discovery of novel properties of well-characterized compounds, as exemplified by statins. These drugs appear to be a particularly interesting group of LLDs because, in light of unorthodox research on the development of therapies for Duchenne muscular dystrophy (DMD) based on statins, their dual nature regarding their effects on skeletal muscle function has been revealed [106]. DMD is the most common and severe form of lethal muscular dystrophy caused by mutations in the dystrophin gene. SIM seems to have a positive impact on the skeletal muscle of dystrophic (mdx) mice, dramatically reducing damage and enhancing their function. These improvements are accompanied by autophagy activation, a recent therapeutic target for DMD, and less oxidative stress [106].

As stated above, models provided insight into the pathogenesis of LLD-induced myotoxicity. The in-vitro studies and research on mammalian model organisms reveal a wide range of data regarding the treatment of diseases induced by LLDs. Despite the many advantages of in-vitro and mammalian models, their research use has some limitations, e.g., results obtained from in-vitro tests do not always reflect in-vivo processes, and in the case of animal models, the number of individuals in the litter does not allow for reliable statistical analysis. This makes the development of new models and further in-depth research necessary.

## 4. Zebrafish Models for Study of Lipid-Lowering Drug-Induced Myopathies

**Figure 1:**
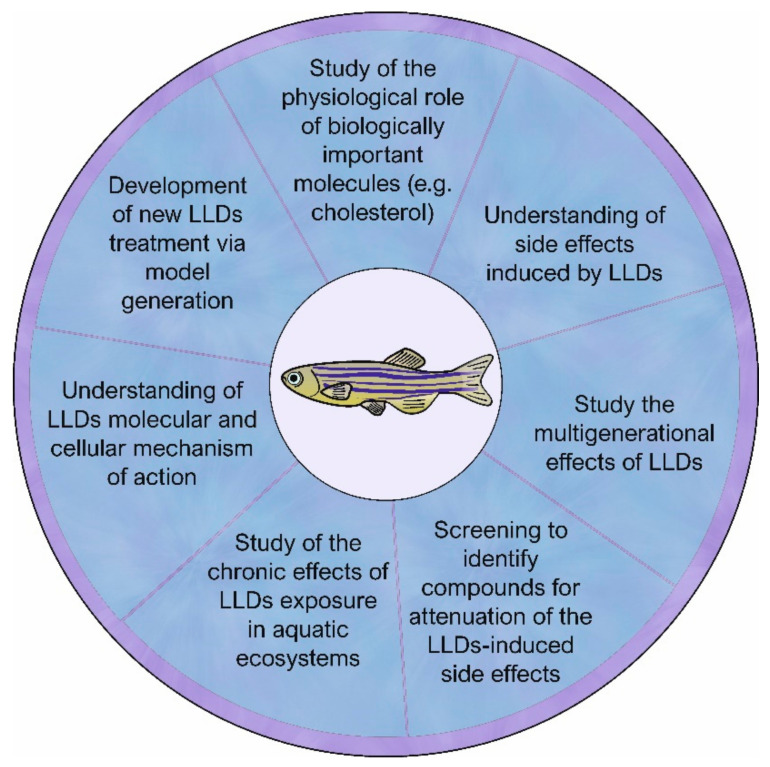
The zebrafish as a model organism in research concerning the mechanism of action of lipid-lowering drugs (LLDs). Zebrafish as a powerful and versatile tool used in broad biological studies can also be successfully used in research regarding lipid-lowering drugs (LLDs). These studies can cover a wide range of topics, including those related to muscle development, LLD-induced myotoxicity, the search for new, safer, and more effective therapies for hyperlipidaemia, and compounds that may prove to be dietary supplements that mitigate the effects of potential LLD-induced myotoxicity. Details can be found in the main text.

Zebrafish has been widely used to study the biological effects of different compounds, including drugs, on the development, myogenesis, muscle tissue structure, and functioning. It has been also shown to be a valid and versatile model of human muscular diseases [107,108,109,110,111]. Research over the past several years also demonstrates that zebrafish has a great potential to be a model for the study of therapeutic side effects, including myopathies, induced by LLDs [112,113,114,115] (Table 1; Figure 1). The widespread use is dictated by its features, favourable from the point of view of scientific research, such as relative handling easiness, the optical transparency of embryos, ex utero development, and the availability of techniques able to assess genetic, physiological, and behavioural changes. Additionally, over the past several years, many research studies validated the use of zebrafish as a model for understanding the mechanisms of action of LLDs. Zebrafish expresses orthologs of different proteins, such as glucocorticoid-induced leucine zipper (GILZ) and HMGCR, which are present in mammals including humans. Studying these proteins is crucial for an in-depth understanding of the mechanism underlying the LLD-induced muscle pathology [113,116]. Also, nuclear receptor (NR)-dependent signalling pathways, involved in the regulation of development, metabolism, and lipid homeostasis, which are possible targets of some lipid-lowering agents, seem to be conserved among metazoans. For example, the orthologs of PPARα, a representative of the NR family, have already been identified in the genome of various vertebrate species including zebrafish [117].

Zebrafish has been useful in making discoveries regarding the ability of statins to act through several diverse mechanisms [113]. Also, statins’ dose-dependent mode of action was demonstrated using zebrafish as a model [114].

Zebrafish can be helpful for the development of new therapeutics with antilipidemic activity, justifying the necessity of generating relevant models based on this organism. For example, zebrafish fed a high-cholesterol diet (HCD) seems to be an effective model to study hypercholesterolaemia [121]. Namely, it was found that treatment with ezetimibe (a lipid-lowering agent) and a combination of low doses of ezetimibe and SIM reduces cholesterol levels in HCD-fed zebrafish larvae [121]. These models may also prove remarkably useful for studying a second issue related to the usage of LLDs: the side effects they cause.

GILZ has been described as an important regulator of, inter alia, skeletal muscle differentiation [122]. The use of the zebrafish, alongside C2C12 cells, primary murine myoblasts/myotubes, and primary human myoblasts, to study statin-induced muscle damage led to the discovery that GILZ plays a key role in mediating the development of this pathological state [116]. Zebrafish statin-provoked GILZ expression results in a series of events, including disruption of embryonic muscle development manifested in impairment of somitogenesis, disrupted embryo tail muscle development, and a reduction of the frequency and dimension of muscle contractions. Also, overexpression of GILZ mimics statin-induced muscle-disrupting effects [116].

In other models GILZ expression is activated by FOXO3 [116]. Whether foxo3b, the zebrafish orthologue of mammalian FOXO3, is engaged in GILZ upregulation-dependent statin-induced muscle damage remains elusive, mostly due to the too broad spectrum of foxo3b morpholino-mediated knockdown effects [116,123].

In zebrafish embryos, as well as in cultured muscle cells, LOV stimulates the expression of atrogin-1 which is a key protein involved in skeletal muscle atrophy and a component of the ubiquitin-proteasome pathway (UPP) [113,124]. LOV treatment of zebrafish embryos leads to morphological changes comprising muscle fibre damage. The phenotype resembles that generated by morpholino-mediated knockdown of zebrafish HMGCR. Inhibition of atrogin-1, the same as forced overexpression of PPARγ coactivator 1α (PGC-1α), a transcriptional coactivator that induces mitochondrial biogenesis, protects both zebrafish embryos and cultured muscle cells from statin-induced muscle damage [113].

Statin-induced inhibition of HMGCR can also be compensated by mevalonate, a precursor of cholesterol, and by geranylgeraniol, a precursor of protein prenylation, but not by farnesol [125]. Inhibitors of the transfer of geranylgeranyl isoprene units to protein targets, such as small GTP-binding proteins, cause atrogin-1 induction followed by statin muscle damage in cultured cells and zebrafish embryos. This observation indicates that small GTP-binding proteins may be necessary to prevent side effects of statin treatment [125].

Studies conducted by Cao [125] led to the conclusion that cholesterol itself is not necessarily the most important factor for statin-induced myotoxicity. The hypothesis that statin-induced side effects are not solely due to reduced cholesterol synthesis, but prenylation, was also supported by other studies using the zebrafish model [126].

SIM influences zebrafish muscle structure and physiology in a dose-dependent manner [127]. High doses lead to more severe muscle structural damage, whereas lower doses cause milder morphological alterations. SIM-induced physiological effects comprise impaired movements and reduced heart beating, which are observed in both low-dose- and high-dose-treated zebrafish embryos. Physiological consequences of SIM treatment are reversible via the addition of exogenous cholesterol. Treatment with this compound leads to a large reduction in cell number. It is also of great significance that SIM, used at different developmental stages, induces varied alterations in zebrafish embryos. At the cellular and subcellular levels, SIM treatment of zebrafish embryos during muscle formation leads to changes in the cytoskeleton (particularly in the intermediate filaments and microfilaments), extracellular matrix, adhesion markers, and myofibril organization [114]. Other studies on zebrafish exposed to SIM confirm that treated embryos display morphological defects accompanied by pericardial oedema [115].

Besides cholesterol, mevalonate is also a precursor of CoQ10, which exhibits antioxidant properties and is a component of the inner mitochondrial membrane, required for oxidative phosphorylation and ATP production. CoQ10 is prenylated; thus its synthesis is inhibited by statins [128]. Reduction of CoQ10 may lead to oxidative stress and mitochondrial dysfunction, which has a particularly negative effect on energy-demanding tissues such as muscle.

Zebrafish larvae exposed to ATV show movement alterations, reduced whole-body tissue metabolism, mostly by modification of oxidative and glycolytic capacities, changed transcriptional activities reflected in increased expression of muscle atrophy markers, and a decreased CoQ10 level [129]. In this way, ATV exposure increased mortality among investigated individuals. ATV-induced decrease in larvae motility and enzyme activities can be reversed with CoQ10 treatment. This provides evidence of a close relationship between the development of myopathic phenotype and declining CoQ10 level [129].

Also, the development of other parts of the musculoskeletal system, such as tendons and ligaments, was investigated through a screen of known bioactive chemicals including statins, in zebrafish. It turned out that inhibition of the mevalonate pathway by statin causes an expansion of the tendon progenitor population. This helped to establish a novel role for the mevalonate pathway in regulating the specification of the tendon lineage [130]. This also confirms that zebrafish is a suitable model for chemical screening for discovering novel genetic pathways involved in developmental processes.

It is worth noting that the zebrafish phenotypes induced by the different statins are not identical. For example, in the study conducted by Thorpe et al. (2004), SIM produced more severe somatic phenotypes than ATV [112]. Embryos treated overnight with either mevinolin (Lovastatin) or SIM (Zocor) exhibited developmental arrest, improper axis elongation, and compressed somites. Embryos treated with ATV exhibited germ cell migration defects and mild morphologic abnormalities [112].

Studies on side effects caused by other groups of compounds belonging to LLDs, such as fibrates, were also conducted using zebrafish. Fibrates, decreasing triacylglycerols and usually increasing high-density lipoprotein concentrations in humans blood, are widely used in the treatment of coronary heart disease [131].

Studies conducted with zebrafish embryos have made it possible to conclude that the fibrates clofibrate and gemfibrozil induce embryonic malabsorption syndrome (EMS), manifested in very little yolk consumption and resulting in small larvae [120,132]. Exposure to clofibrate leads to additional symptoms such as delayed hatching time, induction of higher density, round-shaped neuromuscular junctions, disorganization and less striation of muscular fibres, pericardial oedema, and impairing thyroid gland morphogenesis. A potentially interesting implication of zebrafish LLD-induced side-effect studies is the possibility of using fibrate-induced EMS to study the morphogenetic consequences of impaired nutrient availability during the early stages of vertebrate development [132].

The suitability of zebrafish for multigeneration research also makes it applicable to studies on the side effects of LLDs, including fibrates. The fibrate clofibric acid (CA) exerts an impact on fish lipid metabolism similar to those reported in mammals [119]. CA is an active metabolite of clofibrate, acting as an agonist of PPARα. CA exposure induces a significant reduction in the growth of a parental generation, decreased triglyceride muscle content, and abnormalities in male gonad development with a decrease in the fecundity. It was also established that the expression of the studied genes involved in lipid metabolisms such as *pparaa*, *pparb*, and *acox*, is sex-dependent. Changes in their transcription levels were observed in the parental and offspring generations. Additionally, higher frequency of embryo abnormalities in the offspring of fish exposed to the lowest CA dose was revealed [119].

These considerations seem to support the usefulness of the zebrafish model for studying many aspects related to the need for widespread use of LLDs in humans (Figure 1). This concerns both the mechanisms of action of these drugs together with the study of adverse effects caused by them, as well as generating models that allow the development of new, safer, and more effective therapies to replace the old generation of drugs. The numerous advantages of zebrafish also make it well suited for studying phenomena involving environmental issues and resulting from the massive use of LLDs worldwide.

## 5. Zebrafish Usefulness in Research Dealing with a Lipid-Lowering Drug Environmental Issue

The antilipidaemic contaminant concentrations detected in aquatic environments are increasing among other kinds of pollutions [133,134]. For example, fibrates, frequently reported in wastewater and surface water, are now considered as water pollutants that display a low degradation rate [135,136]. The widespread use of LLDs worldwide makes them a serious threat to the functioning of aquatic ecosystems, especially since some antilipidaemics might display high bioaccumulation potential in aquatic organisms [134,137]. It seems particularly alarming that HMGCR, a target enzyme of statins, displays high sequence conservation, especially within the catalytic binding site, across metazoans [134].

Medicines are bioactive compounds, and thus effective at rather low concentrations. Therefore, even if such compounds are detected in surface waters at trace levels (concentrations from ng/L to μg/L), the environmental risks they pose must be taken seriously [138]. Therefore it will be beneficial to understand the mechanisms underlying the action of antilipidaemic toxicity in aquatic organisms, with fishes as a prime potential target.

Studies on the impact of antilipidemic drugs on the embryologic stages of zebrafish reveal their lethal and sublethal effects at concentrations within ranges found in the environment [139]. The development and survival of fish embryos and yolk-sac larvae depend on the mobilization of yolk lipid constituents. Therefore, the presence in the water of bioactive compounds, able to disrupt lipid metabolism, is a major threat.

Studies that demonstrated adverse effects of various medicines, including statins, on zebrafish embryos such as developmental impairment, and reproduction disturbances, stressed the importance of monitoring the presence of this emerging contaminant in aquatic environments [115,140]. Furthermore, zebrafish embryo/larvae utilization in research regarding statin-induced muscle-damaging effects appears to be justified as they have similar CoQ10 biosynthetic and MVA pathways as those present in mammals [112].

During zebrafish early development, SIM affects the expression of selected NR in an exposure duration-dependent manner [141]. The investigated representatives of the NR family comprise the pregnane X receptor (Pxr), peroxisome proliferator-activated receptor (Ppars), and aryl hydrocarbon receptor (Ahr) [142], which are involved in lipid metabolism, inflammatory responses, immune homeostasis, and xenobiotic metabolism. Conducted studies revealed that short-term SIM treatment of zebrafish embryos upregulates transcription of *ppars*, *pxr*, and *ahr*, whereas long-term SIM treatment results in downregulation of *pxr* and *ahr* with no changes in *ppars* expression. The research results show that the presence of pollutants belonging to antilipidaemics has profound short- and long-term implications for aquatic species’ survival and ecosystem regeneration, especially since statins also display other than cholesterol-lowering activities such as immunomodulatory and anti-inflammatory [143,144].

Zebrafish as a model organism fulfils the need for an assessment of chronic effects of exposure to pharmaceuticals in aquatic ecosystems on non-target aquatic animals. It is also an effective experimental model for conducting aquatic toxicology experiments related to muscle damage induced by LLDs [118]. Zebrafish long-term exposure (90 days) to environmentally relevant SIM concentrations demonstrated that the parental exposure can cause offspring embryonic malformations in a dose-response manner. Assessment of the impact of chronic exposure on adult individuals was conducted using a multi-parametric approach. The assessment involves survival, growth, reproduction, evaluation of biochemical markers (cholesterol and triglycerides) analysis, and examination of the transcription levels of key genes involved in the mevalonate pathway. Measured parameters were significantly impacted by the level of the low-to-intermediate compound, corresponding to environmentally relevant concentrations. Additionally, the sex-dependent differences in obtained results suggest different vulnerability to SIM for females and males [118].

Growing concerns about the release of drugs and their metabolites into the environment have led to the development of new methods that make the extent of contamination monitoring easier. Recently, liquid chromatography coupled to high-resolution quadrupole time-of-flight mass spectrometry (UHPLC-QTOF-MS) was successfully applied for quantification of the levels of statins, fibrates, and their metabolites in the aquatic environments [145].

Research on the environmental impact of antilipidaemics is extremely important, and the development of an effective research methodology and the selection of appropriate experimental models will make it possible to properly assess the risk associated with their mass release into the environment. This is imperative, since it may result in negative repercussions at the level of entire populations, with further impacts on non-aquatic ecosystems, the health of the human population, and economic consequences.

## 6. Perspectives

Current challenges in treatments for hyperlipidaemia are the improvement of so far used therapeutics and development of new, effective and safe compounds. The skeletal muscle-specific HMGCR knockout mice model reveals important information regarding the mechanism of skeletal muscle toxicity caused by statins. The data indicate that the pathological mechanism depends on HMGCR deficiencies and subsequent changes in the level of downstream metabolites of the mevalonate pathway [95]. The authors emphasize that further studies are needed to explain the molecular mechanism responsible for statin-induced myopathy and the function of HMGCR in the skeletal muscles.

Taking into account that zebrafish has become recently a useful research animal model, one can consider it as an additional source of knowledge in the field of drug-induced myopathies. As mentioned above, zebrafish play an important role in drug testing and discovery, e.g., using high-throughput, small molecule screening. Moreover, the comprehensive genomic databases and a growing number of molecular tools for research using zebrafish enable the design of precise genetic experiments. The ease in genetic manipulations of the zebrafish genome, together with the variety of bioinformatic tools, provides the possibility for insightful functional studies, and in-depth interpretation of results [146]. Additionally, it can be considered a convenient model for preclinical evaluation of novel antilipidemic drugs or supplements, especially as the preclinical drug trials are approved e.g., by the FDA (U.S. Food and Drug Administration) [147]. The analysis of molecular data confirms that zebrafish may become a valid research model in functional analysis of mechanisms underlying the statin-induced myopathy mechanism. As mentioned in the previous chapter, the zebrafish mevalonate pathway, disturbed in the statin-induced muscle toxicity, is highly similar to humans [130,148]. Like mice, the zebrafish has orthologs of the human *HMGCR* gene, namely *hmgcra* (gene ID ENSDARG00000052734) and *hmgcrb* (ENSDARG00000105206) [149,150]. The presence of two copies of the gene encoding the protein Hmgcr in zebrafish is probably caused by the expansion of gene repeats in the evolutionary history, possibly facilitated by a population bottleneck [151,152]. However, the identity between sequences of human and zebrafish proteins encoded by these genes reaches 70.52% (BLAST, [153]). Like human HMGCR, the zebrafish protein has hydroxymethylglutaryl-CoA and hydroxymethylglutaryl-CoA reductase activity. Akin to human HMGCR, the zebrafish orthologs have low tissue specificity and are expressed in all anatomical structures [149,154].

There are various resources which can be considered useful in the experiment design. The zebrafish mutants with a point mutation in the *hmgcra* and *hmgcrb* allele, leading to premature stop of translation, are available at the European Zebrafish Resource Center (EZRC) and/or the Zebrafish International Resource Center (ZIRC). The sequence targeting reagents, such as morpholino oligonucleotides, enabling e.g., gene knockout, are also described both for *hmgcra* [113] and *hmgcrb* [155,156,157]. Also, CRISPR-Cas9 (the clustered regularly interspaced short palindromic repeats; Cas9 nuclease) molecular tools are available. The CRISPR-Cas9 molecular system cleaves specific nucleic acid sequences, leading to frame-shift, followed by knock-out of the target gene [158]. The CRISPR-Cas9 approach was shown to be efficient in simultaneous knock-out of two or more genes in zebrafish [146]. The selections of possible target sequences, predicted by the dedicated bioinformatic tool CHOPCHOP, shows multiple possibilities to knock out *hmgcra* and *hmgcrb* with the efficiency reaching 71.7%, and 69% respectively [159]. The applicability of this method is expanding. For example, Kroll et al. (2021) described recently an approach felicitating gene knockout by combining multi-locus targeting with a high mutagenesis rate at each locus. The procedure allows embryos to be converted directly into F0 biallelic knockouts with high efficiency [160].

The unique zebrafish features allow the use of several research techniques that would be incomprehensive with other animal models. The rapid screen of bioactive compounds, able to modulate pathological phenotype and allowing identification of novel drugs or dietary supplements, is one example. Such screens were shown to be successful in drug re-targeting, identifying the FDA-approved drugs as a candidate to e.g., treat Duchenne [161,162] and limb girdle muscular dystrophies [163]. The drug re-targeting approach allows for time and cost-saving, but also opens the possibilities of development of new therapeutic strategies [147]. One of the developing lines of research on the therapy of LLD-induced myotoxicity, in addition to the development of new drugs, should be studies on the therapeutic use of combinations of already known medications with consideration of their proper dosage. This is crucial for greater effectiveness and/or mitigation of side effects of LLD-based treatment. Also in this area, zebrafish appears to be a potentially useful model organism. Studies conducted on a zebrafish model of hypercholesteremia (with HCD-fed zebrafish larvae) showed additive effects of combinations of low doses of ezetimibe and SIM [121]. Moreover, a dose-dependent mode of action of statins has also been demonstrated using the zebrafish [114]. This model also appears to be suitable to conduct comparative studies of adverse effects caused by exposure to various antilipidemics, especially from an environmental perspective [139].

The other example of zebrafish usefulness is based on the fact that the phenotypic changes, especially in the body shape and muscle structure, are easily visible at the early stages of its development. The transparent body and pseudo-crystalline array of the muscle sarcomeres enable observation of polarised light diffraction. The disruption of the regular muscle structure, caused by dystrophy or injury, can be detected in polarised light as a dark area, in contrast to bright, unchanged muscle structure. This simple method, known as birefringence assay, allows for qualitative and quantitative measurement of muscle integrity [164,165]. There are also several microscopic techniques based on the distinct kinds of staining. One such method, based on Evans Blue Dye (EBD), allows for determining the muscle integrity, in particular the muscle membrane instability and damage. The EBD is rapidly taken into degenerating, destroyed, or apoptotic cells, but it is not taken up by cells with an intact sarcolemma. In contrast to mice, the EBD research can be performed in live, intact zebrafish, without the necessity of muscle dissection and sectioning [166,167]. The following example illustrates the possibility of direct visualisation of Ca^2+^ signals in intact zebrafish, which is known to be a crucial signalling molecule in regulations of skeletal muscle development and differentiation, as well as in the LLD-induced myopathies, reviewed in [168].

The medicament treatment can affect the zebrafish muscle mechanical power, and directly change the swimming behaviour. Changes in the zebrafish locomotion kinematics and energetics can also be analysed quantitatively, e.g., using customized software [169,170]. One such methodology, based on the recording of muscle contraction and mathematical algorithms, was applied to quantify the fish internal body force, torque, and power consumption in reaction to neuroactive drugs [170]. The following example is an approach providing a rapid and sensitive functional analysis of zebrafish tail beads. The analysis of visible structures’ movement, correlated with sarcomere length, is possible because of the zebrafish tail transparency [171]. The list of the advanced imaging tracking systems and algorithms allowing for zebrafish behavior assessment are summarized in Table 2.

Zebrafish as a research model is a powerful tool allowing for analysis of LLD induced muscle abnormalities, such as changes in muscle performance, physiology, and structure. As an aquatic animal, it has features which are particularly useful in addressing such important issues as antilipidaemic contaminations in aquatic environments—especially as zebrafish has been shown to be an effective model for assessment of chronic exposure and multigenerational study.

Since zebrafish have many advantages as a model organism, some disadvantages have to be taken into consideration during the experiment planning. The biggest disadvantage of *Danio rerio* as a human disease model is a relative distant phylogenetic relationship with humans, which can result in difficulties in performing pre-clinical trials. Both ex-utero development and small size are considered advantages; however, in some cases this is not so certain. For example, extrauterine development requires chorion removal [175]. The small size is problematic in case of poor water-solubility drugs injection or plasma level of absorbed substances measurements. Therefore, it is difficult to compare the relations between doses used in zebrafish and mammals [146,176]. The animal models do not present all features of particular human disease. Taking under consideration advantages and disadvantages of different animal models, zebrafish is excellent tool to follow-up other, mentioned approaches. It has to be highlighted that connection of studies results obtained from different animal models results in better understanding of human diseases [176] (Figure 1).

The zebrafish, like other animal models of human diseases, usually does not present all features of a particular disease. However, it was shown that zebrafish systems often closely mimic human conditions and complement the data obtained from more established mammalian models [147,164,177]. Additionally, combining the research results obtained using divergent animals allows for the synthesis of gained knowledge.

## Figures and Tables

**Table 1:** List of LLDs in studies using in-vitro and animal models.

Compound	Model	Outcome	Reference
**Statins**			
Atorvastatin (ATV)	in vitro	Decrease of cholesterol level in C2C12 cells, impairment the translocation and function of glucose transporter GLUT4, less myotoxicity, impaired cellular mitochondrial respiration.	[8,32,91]
	zebrafish	*Embryos*: germ cell migration defects and mild morphologic abnormalities.	[112]
	rat	Down-regulation of protein expression (proteins associated with energy production systems (including oxidative and glycolytic enzymes and CK), heat shock proteins, and proteins being components of myofibrils	[101]
Fluvastatin (FLV)	rat	No significant alterations of proteins expression involved in energy production systems, overexpression of chaperonin 60, down-regulation of myozenin 1, FLV high FLV dose -increase the plasma CK content, lower FLV dose—no effects of CK content	[101]
Lovastatin (LOV)	zebrafish	*Embryos:* stimulation of atrogin-1expression, muscle fibre damage, developmental arrest, improper axis elongation, compressed somites	[112,113]
	rat	Skeletal muscle damage	[97]
	goat	High CK activity, myopathy, fibre necrosis, skeletal muscle damage	[105]
	dog	Elevated level of CK, skeletal muscle fibres necrosis	[78]
Pravastatin (PRA)	rat	No change of cholesterol level, small muscle damage, smaller body size	[97]
Rosuvastatin (RSV)	in vitro	Reduction in cholesterol biosynthesis, disruption of muscle cells differentiation and proliferation, changes in profiles of eicosanoids	[92]
Simvastin (SIM)	in vitro	Myotoxicity, impaired cellular mitochondrial respiration, reduction in cholesterol biosynthesis, disruption of muscle cells differentiation and proliferation, changes in profiles of eicosanoids	[91,92]
	zebrafish	*Adults*: muscle structural damage, impaired movements and reduced heart beating, offspring embryonic malformations *Embryos*: changes in the muscle cytoskeleton, extracellular matrix, adhesion markers, and myofibrils organization, pericardial oedema, developmental arrest, improper axis elongation, compressed somites, transcription upregulation of *ppars*, *pxr*, and *ahr*, downregulation of *pxr* and *ahr* with no changes in *ppars* expression	[112,114,115,118]
	rat	Down-regulation PI3k/Akt signalling, and up-regulation FOXO transcription factors, an increase in the transcription of genes implicated in proteasomal- and lysosomal-mediated protein degradation (*MAFbx*), impairment of carbohydrate oxidation, oxidative stress, inflammation, an increased plasma CK level, muscle necrosis	[100]
	rabbit	Necrosis and high serum CK levels, myotonia	[103]
**Fibrates**			
Clofibrate, gemfibrozil	zebrafish	*Embryo*: induction of embryonic malabsorption syndrome (EMS), very little yolk consumption, small-sized larvae, delayed hatching time, round-shaped neuromuscular junctions, disorganization and less striation of muscular fibres, pericardial oedema, impairing thyroid gland morphogenesis	[119]
Clofibric acid	zebrafish	Significant reduction in the growth of a parental generation, decreased triglyceride muscle content, abnormalities in male gonad development with a decrease in the fecundity	[120]
Bezafibrate, ciprofibrate	mouse	Limitation of glucose and three-carbon compounds oxidation enhance fatty acid oxidation in liver cells, muscle disorders	[70]
Fenofibrate	rat	Increase of CK level, muscle damage, influences on glycolytic enzymes	[101]
	dog	Skeletal muscle injury, CK elevated level, skeletal muscle fibres necrosis	[78]
Fenofibrate acid	rat	Inhibition of organic anion transporting polypeptide 1B1 (OATP1B1)	[101]
**Others**			
Ezetimibe	zebrafish	Reduction of CK level in HCD-fed zebrafish larvae	[121]

**Table 2:** List of current methods and techniques for assessing behavior and muscle performance in zebrafish.

Most Recent Methods for Assessing Behavior and Muscle Performance in Zebrafish		
Method	Application	Reference
Measurement of swimming behavior—mathematical and computational analysis	Quantification of the zebrafish larvae swimming behavior and energetics	[170]
Calculating bending moments in swimming fish—experimental data and numerical analysis	Assessment of fish swimming e.g., bending moment pattern, analysis of turning, adult fish swimming at different speeds and accelerations	[172]
Measurement of ultrafast zebrafish larval swimming tail muscles contraction—recording and computational analysis	Measurement of the contractile parameters of the muscle in the larval tail in vivo	[171]
Cell Tracking Profiler (analysis of muscle stem cell responses to injury)—semi-automated image analysis pipeline, based on cell tracking (3D time-lapse datasets)	Accurate measurement of cell shape and movement	[173]
Analysis of stress responses in adult zebrafish—behavioral approach	The analysis of swimming behavior in response to stress, allowing e.g., to examine the pharmacological effects of drugs	[174]

## Data Availability

Not applicable.

## Supplementary Materials

The following are available online at https://www.mdpi.com/article/10.3390/ijms22115654/s1.
