# Supplementary material for: Zebrafish as a Model for the Study of Lipid-Lowering Drug-Induced Myopathies
Source: Int J Mol Sci. 2021 May 26;22(11):5654. doi: 10.3390/ijms22115654 (PMC8198905; doi:10.3390/ijms22115654)
Supplement: Supplementary file 1 [file ijms-22-05654-s001.zip › ijms-1215710-SI.pdf]

Supplementary Table S1

List of lipid-lowering drugs (LLDs) mentioned in the manuscript with their corresponding compound identification number [CID] to access information on their structure and characterization in the PubChem chemical molecule database.

**Compound**	**PubChem compound identification number [CID]**
**Statins**	
Atorvastatin (ATV)	60823
Fluvastatin (FLV)	446155
Lovastatin (LOV)	53232
Pravastatin (PRA)	54687
Rosuvastatin (RSV)	446157
Simvastin (SIM)	54454
**Fibrates**	
Bezafibrate	39042
Ciprofibrate	2763
Clofibrate	2796
Clofibric acid (CA)	2797
Fenofibrate	3339
Fenofibrate acid (FA)	46878853
Gemfibrozil	3463
**Others**	
Ezetimibe	150311
